# Sequence Flow: interactive web application for visualizing partial order alignments

**DOI:** 10.1186/s12864-024-10886-y

**Published:** 2024-10-16

**Authors:** Krzysztof Zdąbłasz, Anna Lisiecka, Norbert Dojer

**Affiliations:** Institute of Informatics, University of Warsaw, Banacha 2, Warszawa, 02-097 Poland

**Keywords:** Multiple sequence alignment, Partial order alignment, Sankey diagram, Webserver

## Abstract

**Background:**

Multiple sequence alignment (MSA) has proven extremely useful in computational biology, especially in inferring evolutionary relationships via phylogenetic analysis and providing insight into protein structure and function. An alternative to the standard MSA model is partial order alignment (POA), in which aligned sequences are represented as paths in a graph rather than rows in a matrix. While the POA model has proven useful in several applications (e.g. sequencing reads assembly and pangenome structure exploration), we lack efficient visualization tools that could highlight its advantages.

**Results:**

We propose Sequence Flow – a web application designed to address the above problem. Sequence Flow presents the POA as a Sankey diagram, a kind of graph visualisation typically used for graphs representing flowcharts. Sequence Flow enables interactive alignment exploration, including fragment selection, highlighting a selected group of sequences, modification of the position of graph nodes, structure simplification etc. After adjustment, the visualization can be saved as a high-quality graphic file. Thanks to the use of SanKEY.js – a JavaScript library for creating Sankey diagrams, designed specifically to visualize POAs, Sequence Flow provides satisfactory performance even with large alignments.

**Conclusions:**

We provide Sankey diagram-based POA visualization tools for both end users (Sequence Flow) and bioinformatic software developers (SanKEY.js). Sequence Flow webservice is available at https://sequenceflow.mimuw.edu.pl/. The source code for SanKEY.js is available at https://github.com/Krzysiekzd/SanKEY.js and for Sequence Flow at https://github.com/Krzysiekzd/SequenceFlow.

## Background

Partial Order Alignment (POA) was proposed in [1] as an alternative approach to multiple sequence alignment (MSA). In POA, the alignment is represented as an acyclic directed graph whose nodes are residues of the aligned sequences, and the directed edges connect successive residues in these sequences. In addition, the set of nodes is divided into clusters equivalent to the MSA columns, with identical symbols from aligned sequences in each cluster combined into a single node. As a result, the aligned sequences form paths in the graph, and the common nodes of these paths are their aligned identical residues.

**Fig. 1:**
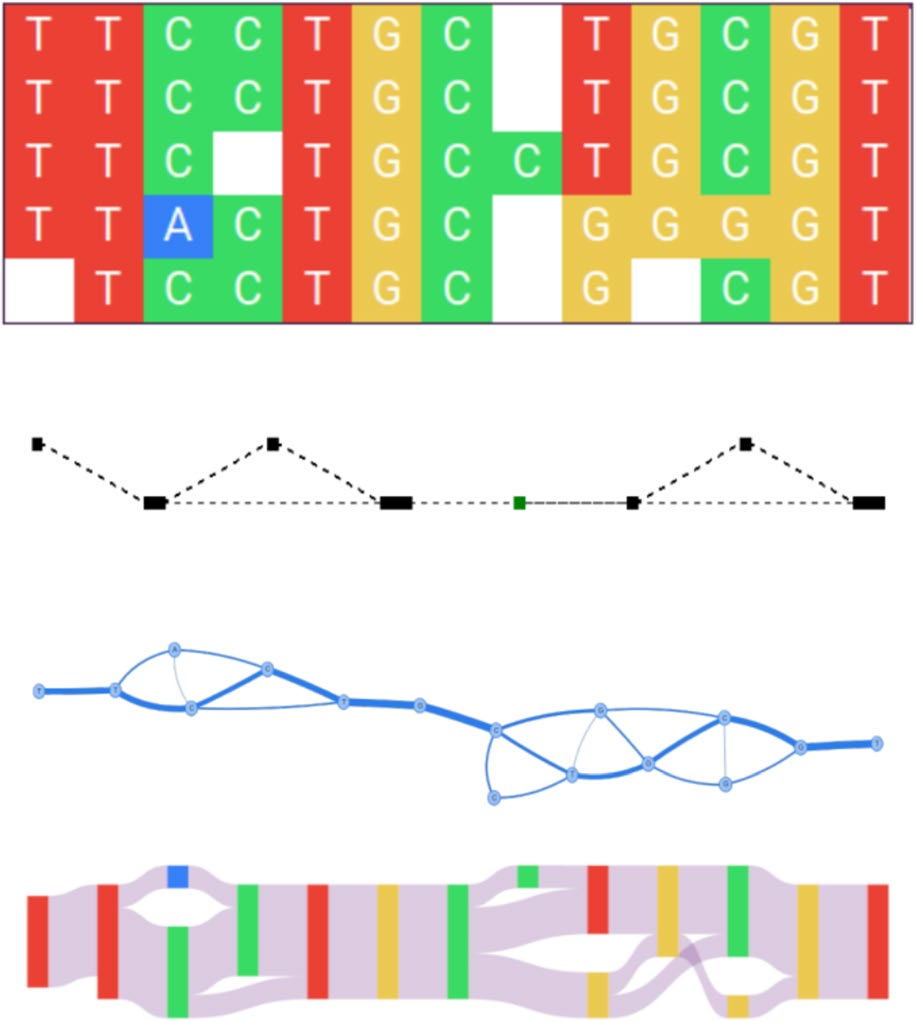
Matrix representation of MSA (top) and the corresponding POA presented using standard graph visualizations (created with poaviz [2] and POAPY [3], respectively) and as a Sankey diagram (bottom)

The first visualization of POA was proposed in [2], but it generates simplified POA graphs. In 2015 J. Dursi proposed POAPY – a simple implementation of POA algorithm for educational/demonstration purposes [3]. POAPY was equipped with an option to create html files with interactive visualizations of full POA graphs. However, as the author points out, the generated visualization stops being useful for graphs with more than a thousand nodes or so. Another POA visualization is included in a PangTreeVis tool [4], but this is designed to present POA graphs being components of pangenome models called *affinity trees*.

Apart from the mentioned limitations of these tools, the presentation of POA using standard directed graph visualization gives moderately satisfactory results. Inspiration for alternative methods can be derived from approaches used to visualize pangenome models, in particular variation graphs. A common feature of POA graphs and variation graphs is that homology between sequences is reflected by shared nodes on the paths representing the sequences. The differences lie in scale and structure - pangenome graphs are often build from whole chromosomes or even entire genomes and must reflect structural variations that violate the acyclicity constraint. Therefore the pangenome visualization tools usually focus on the overall rather than the base-level structure of the graph (e.g. Bandage [5], GfaViz [6] or Sequence Tube Map [7]). The tools that integrate visualization for different scales (e.g. MoMI-G [8] or ODGI [9]) on the nucleotide-scale level use traditional representation.

Another alternative is the *Sankey diagram*, a type of flow chart first used in 1898 to show the energy efficiency of a steam engine [10]. Today, Sankey diagrams are widely used to represent energy flow in physics and engineering [11], and have also been applied to visualize biological data, including regulatory cascades of molecular interactions [12] and time series of gene expression in microbial communities [13]. In such a diagram, each transition is displayed as an arc flowing from the right side of the source node to the left side of the target node. The height of each node and the thickness of each arc is proportional to the flow rate. With vertex placement reflecting the alignment structure, Sankey diagrams can provide a more intuitive alignment visualization than commonly used graphical representations of nodes and edges (see Fig. 1).

**Fig. 2:**
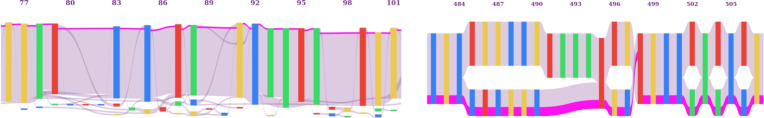
Example POA visualizations created with Sequence Flow. Left: POA representing overlapping long sequencing reads. The consensus path computed to infer the error-corrected read sequence is highlighted. Right: Alternative loci in the MUCIN2 region of the human reference genome GRCh38. The path representing the chromosome 3 reference sequence is highlighted

**Fig. 3:**
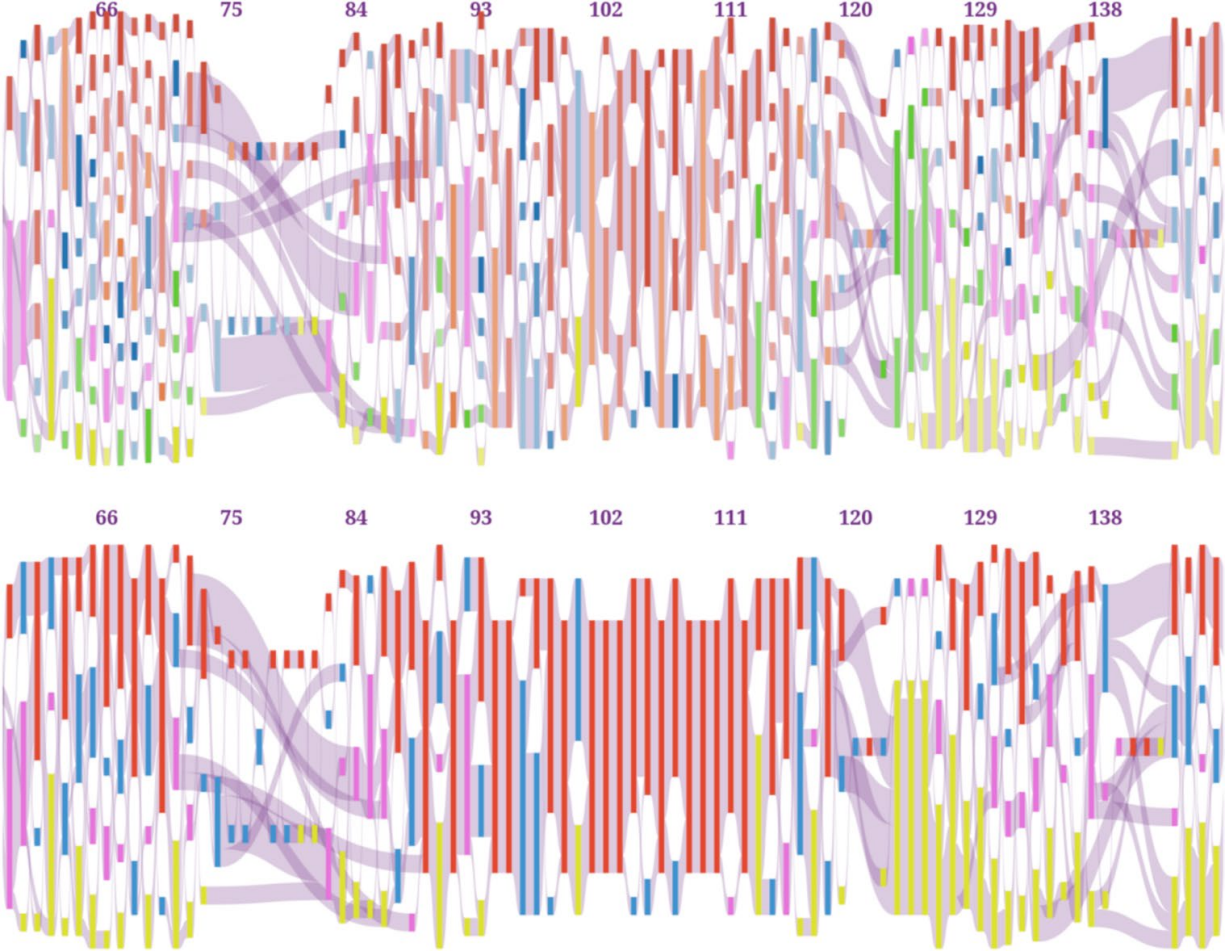
POA graph representing the msl_ref7 alignment from the BAliBASE 2 [14] protein alignment benchmark database (top) and its simplified version (bottom). The latter plot shows the dominance of hydrophobic amino acids in columns 91-116, annotated as the core alignment region

The POA model has proven useful in several applications, e.g. sequencing reads assembly and pangenome structure exploration (see Figs. 2 and 3). Sankey diagram-based visualization can help to understand its advantages and disseminate them to the scientific community. For this purpose, two software tools are presented in this article: *Sequence Flow* and *SanKEY.js*, intended for end users and bioinformatic software developers, respectively.

SanKEY.js is a JavaScript library for creating interactive Sankey diagrams. It has been designed to:

- be free of external dependencies,
- provide a simple, object-oriented API,
- allow a high level of graph customization,
- support interactivity,
- handle huge graphs with tens of thousands of nodes and links in real-time,
- meet the requirements of visualizing POAs, without compromising the overall applicability.

Sequence Flow is a web application for Partial Order Alignment exploration, focusing on Sankey diagram-based POA visualization. It has been designed to be:

- user-friendly – intuitive and available for any browser,
- efficient – prepared to work with limited resources on the client side and running smoothly even with huge alignments,
- customizable – providing users with options to adapt the layout to their requirements,
- integrated with other MSA visualizations and interactive.

## SanKEY.js

### Architecture

**Fig. 4:**
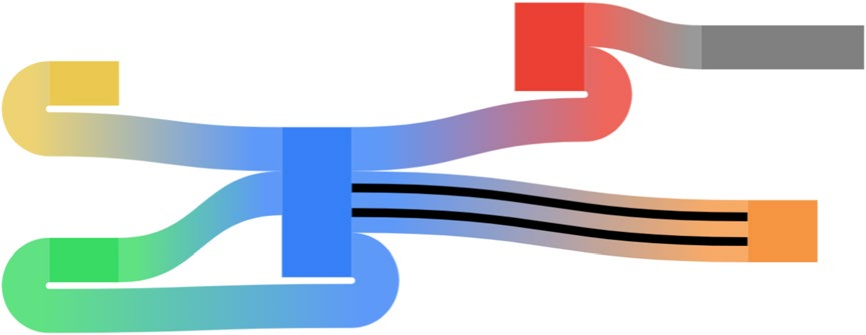
A Sankey diagram illustrating various features of SanKEY.js: nested links (marked with black color), multiple available link shapes, gradient colouring and adjustable node width (grey node)

SanKEY.js requires the user to predefine a sorted list of columns and assign each diagram node to one column. Then, a selected fragment of the diagram can be specified as a range of columns. Consecutive columns within the range are displayed from left to right with uniform spacing. This structure reflects that of POA graphs (diagram columns correspond to MSA columns), but is flexible enough to allow users to specify the desired layout for other applications as well (see Fig. 4). The library is designed to be fully agnostic to the biological context, offering a multi-purpose solution for any data that fits the input format.

### Interactivity

The visualizations created with SanKEY.js are interactive by default and can be easily integrated with the website functionalities. Diagram nodes and links, when hovered over, display a bubble with additional information, the content of which can be modified. Node positions can be adjusted on the vertical axis by moving them with a mouse. Moreover, custom actions can be pinned to events of clicking and hovering plot elements.

### Customization

The appearance of the generated plots can be easily adjusted in the settings. There is a vast range of customization options, including:

- determining the width and height of the nodes,
- defining custom names for the columns,
- displaying/hiding column separators and numbers/names,
- choosing the colours and colour modes of plot elements, and the ability to dynamically modify them.

### Visualizing cycles and bidirected graphs

Typically Sankey diagrams represent directed acyclic graphs, with nodes topologically ordered from left to right and links running from the right sides of source nodes to the left sides of target nodes. SanKEY.js comes with several link shapes that are suitable for displaying also *feedback arcs* (edges having target nodes placed to the left of source nodes) and *reversing joins* (edges connecting two left or two right sides of incident nodes). This enables the visualization of cycles, as well as *bidirected graphs*, which are widely used in pangenomics (see Fig. 4).

### Nested links

The library provides the possibility to specify nested links, e.g. to decompose a link into a number of sublinks connecting the same nodes. Nested links inherit the shape of the parent link, but can be dynamically highlighted to visually present another dimension of the data (see Fig. 4).

### Implementation and performance

The structure of the input data allows SanKEY.js to avoid resource-intensive algorithms for positioning nodes in the diagram. Internal data structures are designed to enable fast rendering and custom graph modifications in real time. Optimization techniques include pre-computing coordinates of plot elements, keeping the information about the longest links, etc.

**Table 1:** Comparison of Sankey diagram generation time using Plotly.js and SanKEY.js libraries

Diagram size		Mean generation time (ms)		
nodes	links	Plotly.js	SanKEY.js	
			initial	reload
10	40	6.33	0.48	0.35
100	400	56.78	3.38	2.81
500	2000	1 129.32	17.82	14.89
1000	4000	7 012.73	38.04	33.00
5000	10000	59 483.17	102.66	93.49
10000	20000	222 179.50	233.26	187.63

Each row presents the mean rendering time on 10 randomly generated graphs. The tests were run on: Chrome 118, Ubuntu 22.04, 12th Gen Intel Core i7-1265U $\times 12$, Mesa Intel Graphics (ADL GT2)

In order to verify the effectiveness of the adopted approach, we compared the performance of SanKEY.js with Plotly.js – the core component of Plotly [15], one of the most popular data visualization platforms. Results presented in Table 1 show that SanKEY.js is $20-1000$ times faster, with the speedup increasing with the size of the dataset.

## SequenceFlow

### Overview

Sequence Flow accepts input MSA files in the most common formats, including fasta, clustal, msf, phylip and stockholm. The alignment is displayed as a Sankey diagram-based POA visualization and in a matrix form. Both representations are aligned with each other, constant column widths are maintained, and nucleotides and amino acids are represented using identical color schemes.

**Fig. 5:**
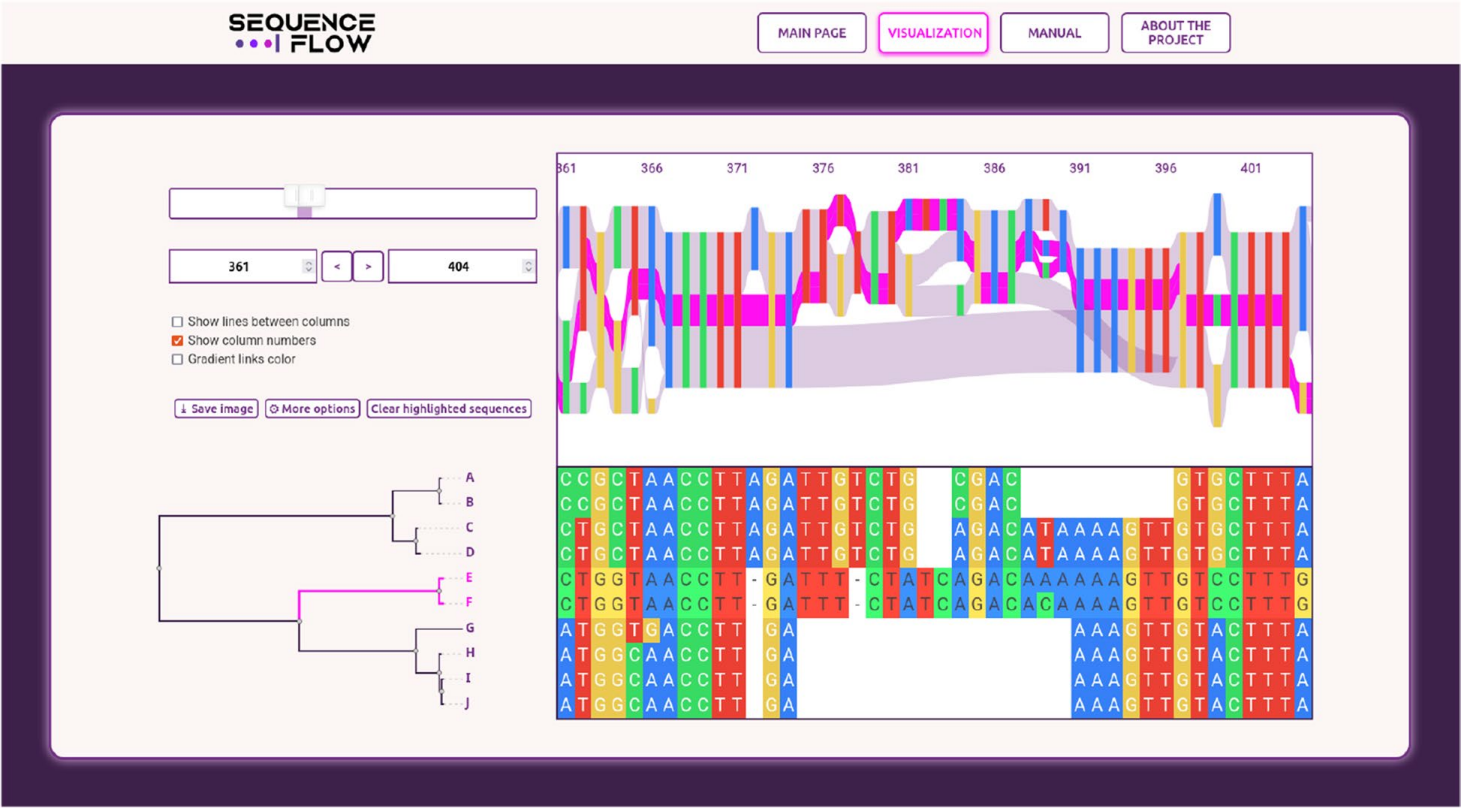
SequenceFlow – screenshot

The user is also able to upload a phylogenetic tree for the aligned sequences in a Newick file. In this case the tree is displayed next to the matrix MSA visualization and the arrangement of both is synchronized: for each aligned sequence, the corresponding tree leaf, sequence identifier and matrix row have the same vertical position coordinate. The main screen contains also a navigation panel, selected options and several action buttons, including the one for saving the current picture in a file (see Fig. 5).

The user can select the displayed fragment of the alignment both by entering the desired range of columns and by moving the currently shown area or its ends. The displayed areas change smoothly and synchronously in both MSA representations.

### Interactivity and sequence highlighting

The POA graph itself represents the alignment in a rather concise way, hiding information about individual sequences. In Sequence Flow, these can be revealed using its interactive features. First, when hovering over nodes and links, information about their underlying sequences is displayed. Second, subgraphs induced by specific sequence subsets can be highlighted and explored. These subsets can be selected interactively through a sequence of the following actions:

- clicking a sequence identifier adds/removes this sequence,
- clicking a vertex or branch of the phylogenetic tree adds/removes all its descendants,
- clicking a POA node adds/removes all sequences passing through this node.

Selected sequences are highlighted synchronously in all three visualization components – as flows in the Sankey diagram, as identifiers in matrix MSA representation and as subtrees of the phylogenetic tree.

### Simplifying the graph

Protein alignments usually demonstrate a much lower level of sequence identity than DNA/RNA alignments. In POA graphs, this translates into a smaller number of common nodes of related sequences and, consequently, a larger number of nodes per column. These factors directly affect the complexity of the POA graph structure and result in difficulty in recognizing and assessing similarity between sequences.

To facilitate the analysis of protein alignments, we provided the option to simplify the graphs. When selected, within each column nodes representing amino acids with similar physicochemical properties are combined (see Fig. 3). By default amino acids are divided into electrically charged, polar uncharged, hydrophobic and special cases (following [16]), but the user can change this division.

### Customization

Apart from selecting the alignment fragment to be displayed, the user can also manually adjust the vertical coordinates of the POA node positions. Moreover, several visualization appearance options are available, including coloring links, showing/hiding column lines and/or numbers, etc. Additionally, the user can change the amino acid grouping scheme, as well as colours assigned to each (group of) nucleotide/amino acid, which are used to display both POA nodes and MSA matrix fields.

### Implementation

Like most modern web services, Sequence Flow leverages a two-layer approach with a separation of responsibilities into back-end and front-end sides.

Server-side utilizes the Python FLASK framework [17], making use of its templating engine and simplicity of developing RESTful APIs. The primary function of the server is to process user-uploaded alignment data, converting it into a standardized format for further use. In addition, the server provides easy access to the collection of example datasets. To facilitate these operations, the application makes use of the popular Biopython library [18], which offers a comprehensive and reliable interface, ensuring robust support for the front-end utilities.

Performance was a top priority when developing the client side of the app, especially considering that visualizing large-scale graphs is resource-intensive. To address this challenge, we utilized pure JavaScript and implemented optimizations in the application’s critical sections. All scripts were minimized to reduce load times and improve overall efficiency. Frequently referenced data, like alignment sequences, are cached on the front-end to limit unnecessary server requests and reduce potential overhead. The POA visualization was implemented using the SanKEY.js library, which serves as the key component of the application. For phylogenetic tree visualization, we leveraged the Phylotree library, ensuring efficient and accurate representation of complex hierarchical data.

## Conclusion

The partial order alignment model has proven useful in several applications, e.g. sequencing reads assembly and pangenome structure exploration. In the current work, we proposed two software tools that can help disseminate Sankey diagram-based visualization of POA to the scientific community:

- SanKEY.js – a JavaScript library for creating interactive Sankey diagrams – is addressed to bioinformatics software developers,
- Sequence Flow – a web application for the exploration of Sankey diagram-based POA visualization – is addressed to end users.

Both tools are designed to be efficient, highly customizable and user-friendly. While Sequence Flow is dedicated particularly to POA graphs, the flexibility of SanKEY.js (e.g. displaying bidirected graphs) makes it suitable to a wide range of applications, including visualizing sequence graphs used in pangenomics.

## Availability and requirements

### Project name: Sequence Flow

Project home pages: https://sequenceflow.mimuw.edu.pl/ (webservice) and https://github.com/Krzysiekzd/Sequence_Flow (source code).

Operating system(s): platform independent.

Programming language: JavaScript, HTML, CSS, and Python.

Other requirements: web browsers, internet connectivity.

License: none.

Any restrictions to use by non-academics: none.

### Project name: SanKEY.js

Project home page: https://github.com/Krzysiekzd/SanKEY.js.

Operating system(s): platform independent.

Programming language: JavaScript.

Other requirements: web browsers, internet connectivity.

License: GNU v3.0.

Any restrictions to use by non-academics: none.

## Data Availability

No datasets were generated or analysed during the current study.

## Abbreviations

POA: Partial Order Alignment
MSA: Multiple Sequence Alignment
API: Application Programming Interface
